# Micro-Mechanical Properties of Slag Rim Formed in Cement–Slag System Evaluated by Nanoindentation Combined with SEM

**DOI:** 10.3390/ma15186347

**Published:** 2022-09-13

**Authors:** Yu Zhang, Minfei Liang, Yidong Gan, Oğuzhan Çopuroğlu

**Affiliations:** Microlab, Section of Materials and Environment, Faculty of Civil Engineering and Geosciences, Delft University of Technology, 2628 CN Delft, The Netherlands

**Keywords:** slag rim, nanoindentation combined with SEM, elastic modulus, hydrotalcite-like phase

## Abstract

Slag rim mainly consists of secondary precipitations such as C–S–H gel phase and hydrotalcite-like phase, which originate from the hydration of slag. In this paper, the micro-mechanical properties of slag rim were characterized by nanoindentation in combination with SEM. It was found that, compared to the C–S–H gel phase, slag rim showed about a 15 GPa higher modulus of elasticity. At the early age, slag cement paste mainly consisted of low-density C–S–H gel phase, high-density C–S–H gel phase, and unhydrated slag particles, as well as calcium hydroxide based on the results of nanoindentation; at the later age, the system contained C–S–H gel phase, slag rim (a mixture of C–S–H gel phase and hydrotalcite-like phase), and unhydrated slag particles.

## 1. Introduction

As a mature addition to the cement industry, slag cement has been used for close to a century in Europe and North America [1,2,3,4]. The current European cement standard EN 197-1 names 27 different cements, 9 of which contain slag as the main component in proportions from 6 to 95 wt.%. In a blended slag cement paste, the hydration of slag depends mainly upon the breakdown of slag structure by OH^−^ ions released from the cement clinker, of which the major reaction is with alkali (Na^+^ and K^+^) hydroxide, and consequently is largely with Ca(OH)_2_. Secondary formations, e.g., C–S–H gel phase and hydrotalcite-like phase, which originate from the hydration of slag, have been identified, and form the ‘inner’ products of slag, also known as the slag rim (the term ‘slag rim’ in the paper does not refer to any specific hydration phase, and it only indicates the location where the secondary precipitations are precipitated) [5].

The characterization of the C–S–H gel phase (in both cement matrix and slag rim) and the hydrotalcite-like phase (in slag rim) in blended slag cement paste have been extensively investigated by various techniques, e.g., thermogravimetric analysis (TGA), X-ray diffraction (XRD), scanning electron microscopy (SEM) equipped with energy-dispersive X-ray spectroscopy (EDS), nuclear magnetic resonance (NMR), etc. [6,7,8,9]. The results obtained from these techniques are mainly related to the chemical properties of hydration products. Meanwhile, the mechanical contributions of various hydration phases formed in slag cement paste have also been frequently reported through nanoindentation [10,11,12], which is a powerful equipment to determine the micro-scale properties of the material microstructure, involving hardness and modulus of elasticity. The micro-mechanical properties of the C–S–H gel phase, portlandite, unreacted cement clinker, and slag particle are thus identified in samples with slag incorporation. Additionally, several studies investigated the micro-mechanical properties of alkali-activated slag systems [13,14,15]. These suggested the presence of three distinct phases with different micro-mechanical properties, namely, pore, unreacted slag particle, and mass gel. Moreover, the properties of mass gel were significantly related to the type of activator and curing age. Additionally, the nanoindentation test has also been explored to characterize the micromechanical properties of other blended cements incorporating silica fume [16,17], fly ash [16,17,18], metakaolin [10], etc., contributing to understanding the microstructural mechanical properties of various cement-based materials.

However, studies on the micro-mechanical properties of slag rim are scarce. This is partially due to the short curing periods in typical laboratory research studies where the slag rim thickness is thin. During indentation tests, only a few perfect indents can be obtained at the slag rim, and thus it is difficult to obtain enough indents to indicate the micro-mechanical properties. Therefore, in this investigation, the micro-mechanical properties of slag rim were determined based on a slag concrete with a service life of about 40 years. It was observed that several decades was sufficient to develop thick rims around unreacted slag grains. In this case, a good number of indents were found to be coincided with the targeted slag rim precisely, without interference from the surrounding materials. The micro-mechanical properties of the slag rim (containing both C–S–H gel phase and hydrotalcite-like phase) were assessed by applying a statistical deconvolution technique on the nanoindentation results. The values obtained in the present paper indicate the micro-mechanical properties of slag rim for the first time, and could further contribute to understanding the mechanical performance of slag-rich concrete, especially at a later age.

## 2. Materials and Methodology

### 2.1. Sample Information

Two specimens were prepared for the experimental investigation in this paper, representing early- and later-age samples, respectively. The first one (named as sample A, the early-age sample) was made from CEM I 42.5 N (manufactured by ENCI Maastricht B.V.) and slag. The slag cement paste was casted with sealed curing in the laboratory at 20 ± 3 °C. The slag to cement ratio was 7/3 by mass (considered as CEM III/B), and the water to binder ratio was 0.4. The chemical composition of the raw materials as determined by XRF is presented in Table 1. Measurements of sample A were carried out after 3 months of curing. The second one (named as sample B, the later-age sample) was a slag concrete sample collected from a wind deflection screen near Calandbrug, Europoort Rotterdam (Port of Rotterdam), Netherlands, which was built in 1985. The cement type used was reported as CEM III/B.

### 2.2. Experimental Methods

Crushed samples were immersed in isopropanol solution to stop hydration. In order to obtain a very flat and smooth surface for nanoindentation test, samples were ground with #180, #220, #320, #800, and #1200 SiC grinding papers cooled with pure ethanol sequentially, and polished by 9, 3, 1, and 0.25 μm diamond paste in turn. After each step, the sample was immersed in an ultrasonic bath filled with 100% ethanol for 30 s for cleaning the dust and diamond particles left on the surface.

To obtain the micro-mechanical properties of slag rim accurately, a method combining SEM and nanoindentation was employed in the study. At first, the nanoindentation test was performed on the sample surface. Then, FEI QUANTA FEG 650 ESEM was employed to locate the exact indentation points through which each indent and the corresponding phase underneath the indent were identified. A similar process was also conducted in the work of [15,19]. The distance between individual indents was 40 μm. Around 800 indents in total were performed on each polished sample.

The basic principle of nanoindentation is to press a tip with known properties into a material at a small scale. Agilent Nano Indenter G200 (Keysight, Santa Rosa, CA, USA), equipped with a Berkovich tip was used in the present paper. The Continuous Stiffness Method (CSM) developed by Oliver and Pharr [20] was adopted to evaluate the indentation modulus E of different phases. This method superimposes a small oscillation on the primary loading signal and analyzes the response through a frequency-specific amplifier. It allows for the continuous measurement of contact stiffness throughout the indentation depth, which was set to 2000 nm in the study. Micro-mechanical properties are thus obtained as a function of penetration depth.

**Figure 1:**
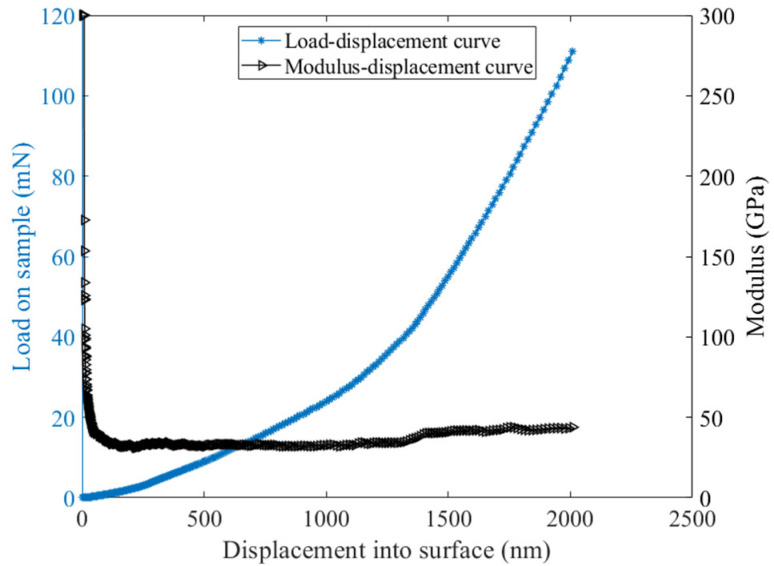
A typical load–displacement curve and the corresponding modulus–displacement graph generated from the loading history.

Figure 1 shows a typical load–displacement curve and the corresponding modulus-displacement graph generated from the loading history. It was noted that after loading when the indenter contacted the sample surface, the measured modulus first decreased rapidly and then stabilized at a certain value upon reaching a specified penetration depth. The indentation elastic modulus was averaged from 1000 to 2000 nm in all measurements. Meanwhile, one needs to keep in mind that due to the close mixture of hydrates in the matrix, the derived modulus for each indent is actually an average mechanical response of the interaction volume underneath the indenter. The micro-mechanical properties of both the indented materials and materials surrounding the indent with the length scale of 3–5 *h_max_* (maximum indentation depth, 2000 nm in this paper) are encompassed [21,22,23].

The indentation modulus of elasticity and hardness are defined by the following equations [24]. These are related to the contact stiffness S, the projected indenter area $A_{c}$, the peak load $P_{max}$, and a geometric constant β, which is equal to 1.034 for the Berkovich tip.

$$E=\frac{dP}{dh}=\frac{\sqrt{\pi }}{2\beta }\frac{S}{\sqrt{A_{c}}}$$

$$H=\frac{P_{max}}{A_{c}}$$

The effect of a non-rigid indenter can be accounted for by the following equation:

$$\frac{1}{E}=\frac{1-v_{s}^{2}}{E_{s}}+\frac{1-v_{i}^{2}}{E_{i}}$$

where $E_{s}$ and $v_{s}$ are the elastic modulus and Poisson’s ratio of the sample, respectively. In the cement–slag systems, Poisson’s ratio was assumed to be 0.20 for all measurements. $E_{i}$ and $v_{i}$ are the elastic modulus and Poisson’s ratio of the indentation tip, respectively ($E_{i}$ = 1141 GPa, $v_{i}$ = 0.07).

For thermogravimetric analysis, the hydration of the sample was also stopped by solvent exchange with isopropanol. Slices cut and selected from samples were ground and meshed to below 63 μm. TGA was performed on Netzsch STA 449 F3 Jupiter under an argon atmosphere. About 40 mg of sample powder was heated from 40 to 800 °C with a heating rate of 10 °C/min in an Al_2_O_3_ crucible with an identical crucible as the reference.

For microanalysis, the sample was examined by FEI QUANTA FEG 650 ESEM under back-scattered electron (BSE) mode equipped with an EDS detector. All microanalyses were carried out at an accelerating voltage of 10 kV and a working distance of 10 mm, respectively.

### 2.3. Analytical Methods

Statistical deconvolution is frequently used to analyze the large amount of data produced by grid indentation [12,14,25,26]. Cementitious composites are known as heterogeneous systems containing components such as pores, hydration products, and unreacted binder particles at the microscale. This kind of system can be recognized as a superposition of different components, where each component has its own state that can be described by a probability density function. Therefore, the superposition of these components (or states) can also be represented as a probability density function that contains both the probability distribution of each component and the probability of it being that component amongst the others. A Gaussian mixture model (GMM) was applied in the paper, and it was assumed that the probability density function was Gaussian and the superposition of these components was linear [27].

The vector X consists of all components, $X_{j}$, where j∈{1, 2, …, k}. GMM describes the superposition of each component as a density function that equals the sum of joint distributions of a measured value being a particular component as well as the distribution of each component itself, where

$$p\left(X|\left\{w,\;\mu ,\;\sigma^{2}\right\}\right)=\overset{k}{\underset{j=i}{\sum }}w_{i}p\left(X_{j}|\left\{\mu_{j},\;\sigma_{j}^{2}\right\}\right)$$

$w_{j}$ is the probability of being a particular component $X_{j}$ and the sum of it ($w_{1},\;w_{2},\;\ldots ,\;w_{k}$) should be equal to 1, i.e.,

$$\overset{k}{\underset{j=1}{\sum }}w_{j}=1$$

The probability of a particular component $X_{j}$ is described by a Gaussian distribution with its mean $\mu_{j}$ and variance $\sigma_{j}^{2}$. Given the number of components k, these mentioned parameters $w_{j}$, $\mu_{j}$, $\sigma_{j}^{2}$ can be fitted sequentially with the following equation.

$$p\left(X_{j}|\left\{\mu_{j},\;\sigma_{j}^{2}\right\}\right)=\frac{1}{\sqrt{2\pi \sigma_{j}^{2}}}exp\left(\frac{{\left(X_{j}-\mu_{j}\right)}^{2}}{2\sigma_{j}^{2}}\right)$$

## 3. Results

### 3.1. Subsection Hydration Products

**Figure 2:**
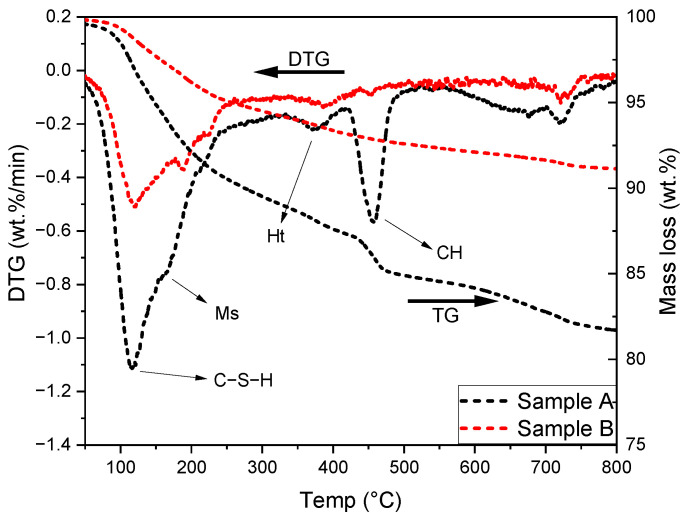
TG and DTG results of samples A and B. Ms: calcium monosulfoaluminate; Ht: hydrotalcite-like phase; CH: portlandite.

The TG and DTG results of these two samples are shown in Figure 2. The main hydration products formed were similar to each other. The peak at 100–150 °C suggested the presence of the C–S–H gel phase. Moreover, the shoulder at ~200 °C implied the formation of calcium monosulfoaluminate, originated from the transformation of ettringite with time [28]. Additionally, the peaks located at approximately 350 °C indicated the formation of the hydrotalcite-like phase with the provision of MgO and Al_2_O_3_ from slag [9]. The peak between 400 and 500 °C was sourced from the dehydroxylation of portlandite [6]. It was distinct in sample A at 90 days, while it almost disappeared in sample B after around 40 years of hydration. It is worthwhile to mention that the results of sample A and B cannot be compared directly as a certain number of sands were included during the powder preparation of sample B, inevitably.

**Figure 3:**
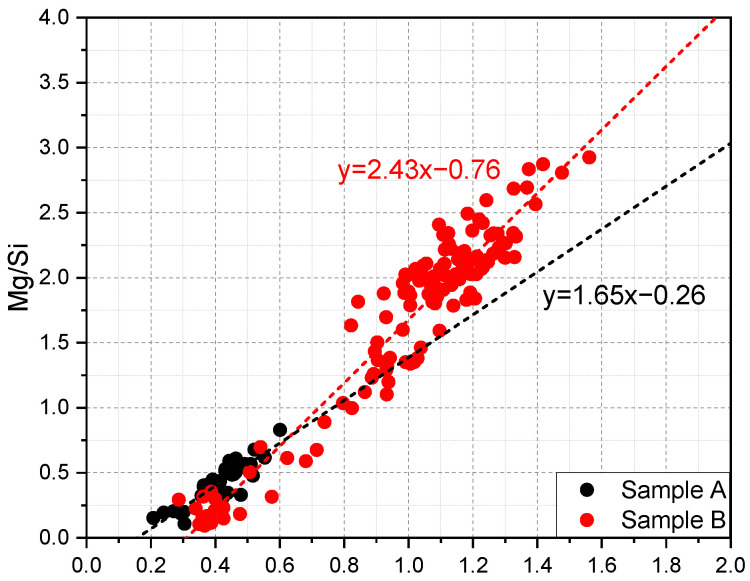
Scatter plot of Mg/Si vs. Al/Si in the molar ratio of samples A and B.

As mentioned, the ‘inner’ products of slag, forming the rim around unreacted slag grain, are a binary mixture of the C–S–H gel phase and the hydrotalcite-like phase. Generally, the 2D scatter plot of EDS points can be interpreted to give the compositions of phases present and the intermixing between them. Moreover, the slope of the regression line fitting the scatter plot of Mg/Si vs. Al/Si in the molar ratio indicates the Mg/Al atomic ratio of the hydrotalcite-like phase. Figure 3 reveals the scatter plot of Mg/Si vs. Al/Si of sample A and B. In sample A, most scatter points were characterized by Mg/Si < 1.0. As for sample B which had been hydrated for decades, a fair number of EDS points were distributed at Mg/Si ≈ 2.0. In other words, the C–S–H gel phase prevailed in the ‘inner’ products of slag in sample A, while the hydrotalcite-like phase dominated in the rim of slag in sample B.

### 3.2. Microstructure Characterization

**Figure 4:**
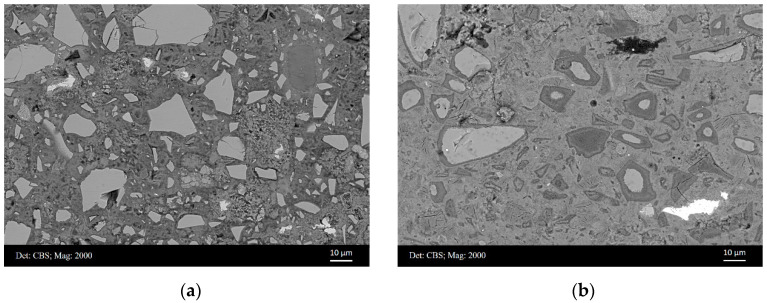
Representative BSE micrographs of (**a**) sample A and (**b**) sample B, respectively.

Figure 4a,b illustrate the typical microstructure of sample A and B, respectively. A mixture of unhydrated slag grains, hydrated phases, and pores were observed to form the microstructure. Mg–Al-rich hydrates were noticed as the precipitates in the slag rims, which displayed a relatively dark coloration because of their low mean atomic number. However, the thickness of slag rim was very thin in sample A at this early age (Figure 4a). On the other hand, it can be seen that the rims were quite thick in sample B at later age (Figure 4b). Occasionally fully hydrated slag grains were observed in the matrix of sample B.

**Figure 5:**
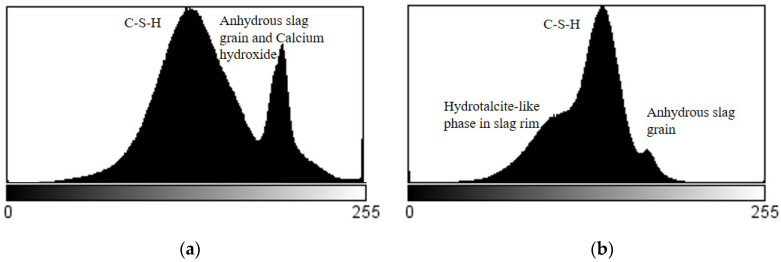
Grey value histogram of images shown in Figure 4, representing (**a**) sample A and (**b**) sample B, respectively.

In general, the higher the mean atomic number of a phase, the higher the pixels generated, and consequently the brighter the phase will appear in the BSE image [29]. Figure 5a,b indicate the grey value histograms of the images shown in Figure 4. It was evident that two peaks occurred in the grey value histogram of sample A, representing the C–S–H gel phase and anhydrous slag grain as well as calcium hydroxide (the peaks for anhydrous slag grain and calcium hydroxide were overlapped with each other [29]), respectively. Note that low-density C–S–H gel phases and high-density C–S–H gel phases cannot be distinguished by grey level. They overlapped into the single peak seen in Figure 5a. In sample B, three phases could be discriminated, i.e., the hydrotalcite-like phase, the C–S–H gel phase, and anhydrous slag grain. The C–S–H gel phase accounted for the majority of the matrix, and was the most frequently observed. Furthermore, it was noted that the proportion of anhydrous slag grain plus calcium hydroxide decreased considerably with proceeding hydration, increasing the amount of hydrotalcite-like phase as a result.

**Figure 6:**
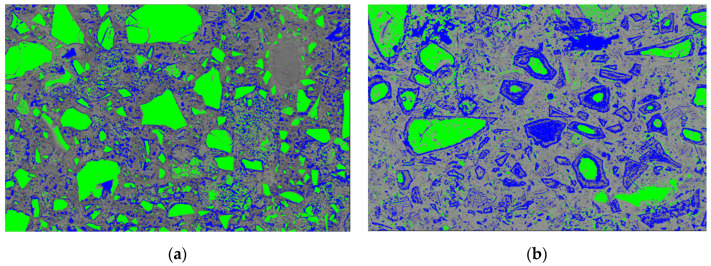
The distribution of phases in (**a**) sample A and (**b**) sample B, respectively, i.e., 0–100 (blue, hydrotalcite-like phase in slag rim and a small fraction of pore), 100–160 (origin, C–S–H gel phase), and 160–255 (green, anhydrous slag grain as well as calcium hydroxide).

Phase segmentations were also carried out by thresholding grey pixel values (0~255) based on the generated histograms. The range of pixel values of the three segmented phases were 0–100 (blue), 100–160 (origin), and 160–255 (green), and are highlighted with artificial colorations in Figure 6a,b. In principle, high grey value (160–255) mainly corresponded to anhydrous slag grains, calcium hydroxide, and minor unhydrated cement clinkers. The hydrotalcite-like phase in the rim of the slag presented relatively low grey value (0–100), owing to its low mean atomic number. The grey value of the pore was also in this range; however, its fraction was very small (see Table 2). The C–S–H gel phase accounted for the majority of the matrix displaying medium grey value (100–160). The distribution of different phases in the microstructure was thus identified visually. Again, note that a rich accumulation of hydrotalcite-like phase in the rim of slag can be observed in sample B, which was almost invisible for sample A. 

The surface/volume fraction of each phase was then determined through BSE image analysis, which allowed different phases to be identified according to their corresponding grey values [29]. More than 10 randomly chosen BSE images of each sample at a magnification of ×500 were acquired under the same condition. The fraction of each phase was calculated using the software ImageJ subsequently, and the results are listed in Table 2.

Apparently, the proportion of the hydrotalcite-like phase of sample B was significantly higher than that of sample A, because of the continuous hydration of unreacted slag particles. This was also reflected by the thickness of slag rim around the unhydrated slag particle, as observed in Figure 4. Moreover, it is worthwhile to mention that not only the hydrotalcite-like phase but also the C–S–H gel phase was formed within the rim of slag. Thus, the actual surface/volume fraction of ***slag rim*** should be greater than that of the hydrotalcite-like phase, especially for sample B, where a large amount of C–S–H gel phase was formed in the slag rim (Figure 6b). Therefore, indents had more chance to be located at the slag rim exactly when measuring sample B, while few indents could be found to detect micro-mechanical properties without interference for sample A.

### 3.3. Micro-Mechanical Properties of Slag Rim

#### 3.3.1. Nanoindentation Combined with SEM-EDS

**Figure 7:**
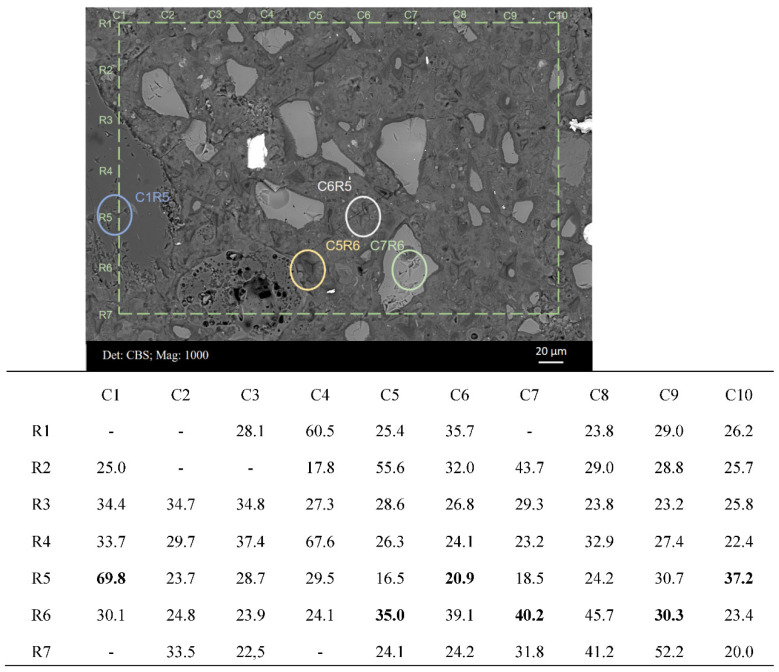
BSE micrograph of one representative area of sample B with indents and the corresponding values of indentation modulus (GPa).

Figure 7 displays the BSE micrograph of a typical area from sample B after the nanoindentation test. The measured modulus values are shown in the table below. Some of the indents were easily located and are shown (circled) in the figure. For example, indents C7R6 and C1R5 were positioned at an unhydrated slag particle and aggregate, respectively. Most indents were located within the cement matrix, e.g., indent C6R5 presenting an elastic modulus of 20.9 GPa. This value was in agreement with the measured values of the C–S–H gel phase in the reported studies [30]. Meanwhile, several indents measured slag rims around partially hydrated slag grains (indents C9R6 and C10R5) and fully hydrated slag grains (indent C5R6). It was noticed that the indentation modulus of slag rim, in the range of 30~40 GPa, was higher than that of the C–S–H gel phase, which commonly fluctuated at around 20 GPa [30].

**Figure 8:**
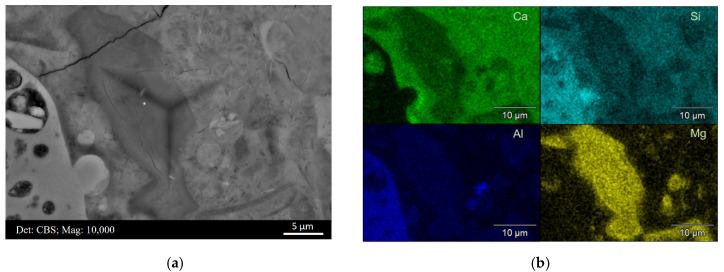
(**a**) BSE micrograph of indent C5R6 located at the hydration products of slag grain exactly and (**b**) the main element mappings of this area.

Figure 8a,b display a BSE micrograph covering indent C5R6 and the main element mappings (Ca, Si Al, and Mg) of this area, respectively. This area can be assigned to a completely hydrated slag grain, and thus indent C5R6 measured the micro-mechanical properties of slag ‘inner’ products exactly. As shown in Figure 8b, magnesium appeared to be distributed in the original slag grain region entirely and could not diffuse into the matrix. Moreover, this region appeared relatively dark with respect to calcium and silicon, which implied that most Ca and Si had migrated out into the cement matrix. Therefore, the hydrotalcite-like phase prevailed in this area belonging to slag grain originally.

Moreover, one needs to keep in mind that due to the mixture of hydrates within slag rim, the derived indentation modulus of each indent is actually an average mechanical response of the interaction volume underneath the indenter. Therefore, the micro-mechanical properties of slag rim are definitely related to, e.g., the molar ratio of the C–S–H gel phase to the hydrotalcite-like phase and the spatial distribution of them underneath each indenter.

#### 3.3.2. Frequency Histogram of Indentation Modulus and Hardness

**Figure 9:**
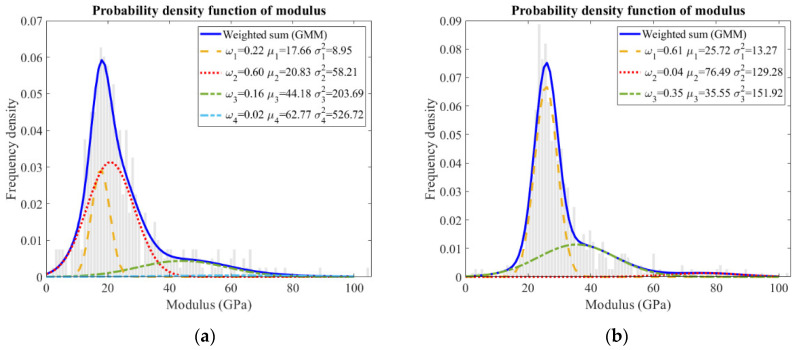
GMM results for the indentation modulus of (**a**) sample A and (**b**) sample B from grid indentations.

The relative frequency of indentation modulus obtained from the sample is plotted in Figure 9. During the measurement, two or three representative regions were selected for the nanoindentation tests. Around 800 indents were designed on a grid to ensure that the results were reliable and representative. Moreover, a Gaussian mixture model was applied to deconvolute the experimental data, and the individual mechanical values were assigned to the most probable components.

Statistically, the highest peak in the frequency histogram was supposed to be the C–S–H gel phase. This peak was narrow with a low variance in sample B, corresponding to an indentation modulus of approximately 25.72 ± 3.64 GPa for it. The stiffness value was found to be reasonable as most calcium hydroxide was consumed (Figure 2), and low-density as well as high-density C–S–H gel phase merged into one homogenous gel phase after such a long hydration time. Comparatively, two C–S–H gel phases were distinguished in sample A, i.e., 17.66 ± 2.99 GPa and 20.83 ± 7.63 GPa, corresponding to the low-density and high-density C–S–H gel phases, respectively. The peak regarding the high-density C–S–H gel phase was broader, which was probably overlapped with neighboring phases, including the low-density C–S–H gel phase [25,31,32] and calcium hydroxide [33].

Another significant feature was the occurrence of a shoulder following the main Gaussian peak in the frequency plot of sample B, indicating the slag rim according to the inspections on the indentation locations and the corresponding phases (in Figure 7). The indentation modulus of the slag rim was 35.55 ± 12.33 GPa. The shoulder was also broad, incorporating the values representative of unreacted slag particles and the C–S–H gel phase. When loading at the slag rims, the interference from unhydrated slag particles and cement matrix emerged, resulting in a broad peak. Nonetheless, about a 15 GPa higher modulus of elasticity was obtained for the slag rim compared to that of the C–S–H gel phase. Considering that the slag rim was a binary mixture of the C–S–H gel phase and the hydrotalcite-like phase, and the indentation modulus of the C–S–H gel phase commonly varied at around 20 GPa, it was concluded that the indentation modulus of the hydrotalcite-like phase was larger than roughly 40 GPa.

**Figure 10:**
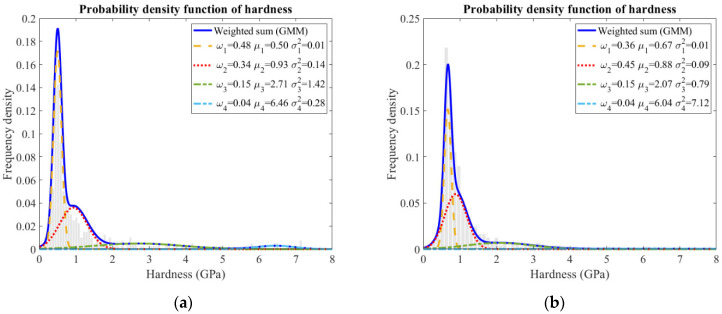
GMM results for the hardness of (**a**) sample A and (**b**) sample B from grid indentations.

As for hardness, which is related to the yield strength of local materials, the response was similar in these two samples (Figure 10). The highest peak (H < 1 GPa) was assigned to the (low-density) C–S–H gel phase [30]. As for the shoulder (close to 1 GPa) following the main Gaussian peak, it can be assigned to the high-density C–S–H gel phase in sample A (Figure 10a); however, it belonged to slag rim in sample B (Figure 10b). Representative indents from Figure 7 and the corresponding hardness values are listed in Table 3.

Collectively, based on the results of the nanoindentation test, it can be stated that at early age, slag-rich cement paste mainly consists of low-density C–S–H gel phase, high-density C–S–H gel phase (a strong overlap with low-density C–S–H gel phase and calcium hydroxide), and unhydrated slag particles and calcium hydroxide from a mechanical viewpoint. However, it contains C–S–H gel phase, slag rim (a mixture of C–S–H gel phase and hydrotalcite-like phase, overlapped with C–S–H gel phase and unhydrated slag particle), and unhydrated slag particles for later-age samples.

## 4. Discussion

The hydrotalcite-like phase, as the main precipitation of pozzolanic reaction between slag and portlandite, is closely intermixed with the C–S–H gel phase, forming the so-called ‘inner’ products of slag within the original slag region. Normally, studies performed on slag cement or alkali-activated slag pastes in the laboratory are mostly cured for several months to one year. Although the hydration degree of slag can reach 30–40% within this time scale [34,35], the majority of this is sourced from the hydration of very small slag grains. The thickness of slag rim around unhydrated slag particles is still very thin at this stage (e.g., Figure 4a). Considering the interaction volume underneath the indenter (2000 nm in depth and 6000–10,000 nm in length in the paper), few indents can be located exactly at slag rims or totally hydrated slag grains, which was the case of sample A in the present paper. This also explains why no investigations study the micro-mechanical properties of slag rim in slag cement or alkali-activated slag pastes.

**Figure 11:**
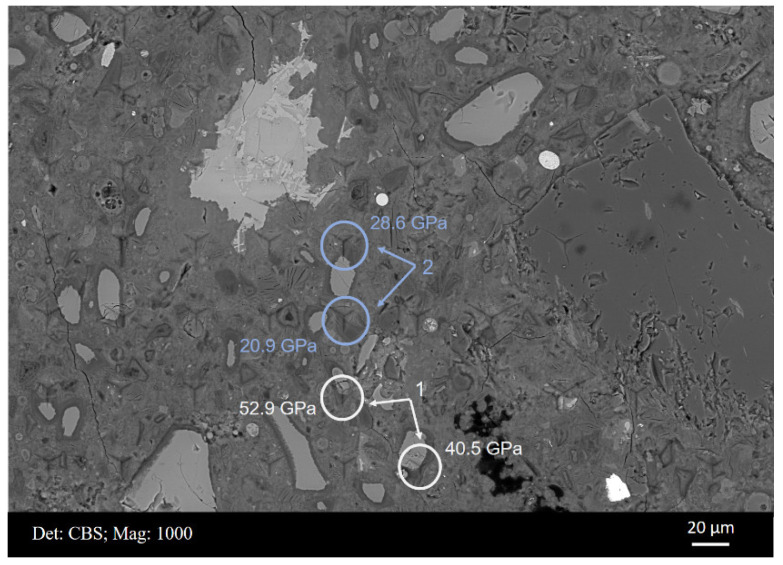
Indents with the interference either from unhydrated slag particles or cement matrix.

With the proceeding hydration, the rims around unreacted slag particles become thicker, and totally hydrated slag grains at a size of >10 μm are also frequently observed (Figure 4b and Figure 11). Under this circumstance, indents have more chance to be positioned at the slag rim. This is exhibited clearly in Figure 7, where indents can be seen to be targeted at slag rims around partially hydrated slag grains (indents C9R6 and C10R5) or fully hydrated slag grains (indent C5R6).

Figure 11 illustrates some cases demonstrating that indents are partially located at slag rims with the interference either from unhydrated slag particles or the cement matrix. Indents in Circle 1 illustrated the interference from unreacted slag particles when the rims were not thick enough. A relatively higher modulus was obtained compared with that located only at the slag rim. On the contrary, the indents in Circle 2 revealed interference from the cement matrix, and a relatively lower modulus was measured. These cases also explained why the shoulder assigned to the slag rim was broad (Figure 9b).

This significant difference in the elastic modulus between the C–S–H gel phase and the hydrotalcite-like phase also indicated the variations in chemical composition, nanocrystalline structure, etc., between them. By means of transmission electron microscopy (TEM), the works in [9] revealed that the fine fibrillar-like C–S–H gel phase intermixed with the lath-like hydrotalcite-like phase in the rim of slag. At an atomic level, the hydrotalcite-like phase is also referred to as layered double hydroxides (LDHs). The brucite-like trioctahedral layers present positive charge due to the isomorphic substitution of Al^3+^ for Mg^2+^. The central Mg^2+^ ion is surrounded by six hydroxyl groups in an octahedral configuration. Through edge-sharing, these octahedral units form infinite, charge-neutral layers, and these layers then stack on top of one another to form a three-dimensional structure [36,37,38]. As for the C–S–H gel phase, it elongates along one direction that results in a fibrous structure [7,39]. Comparatively, the infinitely stacked layer structure of the hydrotalcite-like phase is capable of withstanding larger deformation than the linear silicate chain structure of the C–S–H gel phase, thus presenting a higher modulus.

Naturally, it is concluded that the higher the hydrotalcite-like phase content, the higher the indentation modulus of slag rim. Thus, this raises the question of how to obtain as much hydrotalcite-like phase as possible after slag hydration. Its formation is directly associated with the MgO content of raw slag. The work in [40,41] confirmed that the higher MgO content in slag and the higher amount of hydrotalcite-like phase produced after hydration.

It is well accepted that pore refinement originates from the pozzolanic reaction, and this explains the good mechanical property of slag-rich cement or concrete at later age. However, the results obtained in this paper provide a new aspect to understand it. With the continuous hydration of slag grain, more hydrotalcite-like phase is produced, and thus a new component (i.e., slag rim) with a higher elastic modulus starts to stand out in the system, which also contributes to the enhanced mechanical performance of slag-rich cement at later age. Moreover, the results in this paper can improve the accuracy of mechanical simulations of slag-containing systems as none of the existing models take slag rim as an independent component into consideration.

## 5. Conclusions

The authors in the present paper employed nanoindentation and SEM techniques together to investigate the micro-mechanical properties of slag rim formed in a cement–slag system. The main conclusions drawn were as follows:

(1) First, ~15 GPa higher modulus was obtained for slag rim compared with that of the C–S–H gel phase. Considering that the modulus of the C–S–H gel phase commonly varied around 20 GPa and it was in the range of 30~40 GPa for slag rim, it was concluded reasonably that the indentation modulus of the hydrotalcite-like phase was larger than ~40 GPa roughly.
(2) At early age, slag cement paste mainly consisted of low-density C–S–H gel phase, high-density C–S–H gel phase, and unhydrated slag particles, as well as calcium hydroxide; at later age, the system contained C–S–H gel phase, slag rim (a mixture of C–S–H gel phase and hydrotalcite-like phase), and unhydrated slag particles.

## Figures and Tables

**Table 1:** Chemical compositions (wt.%) of cement and slag used for sample A as determined by XRF.

	CaO	SiO_2_	Al_2_O_3_	MgO	FeO/Fe_2_O_3_	TiO_2_	MnO/Mn_2_O_3_	Na_2_O	K_2_O	SO_3_	Residual
Cement	64	20	5	-	3	-	-	0.58	-	2.93	4.49
Slag	37.04	37.79	14.51	8.83	0.28	0.70	0.17	0.24	0.25	0.01	0.18

**Table 2:** The fraction of each phase determined by ImageJ based on their corresponding grey values (%).

Phase	Pore	Hydrotalcite-Like Phase	C–S–H Gel Phase ^1^	Anhydrous Slag Grains ^2^
Grey Value	0~50	50~100	100~160	160~255
Sample A	0.91 ± 0.25	8.62 ± 1.32	47.34 ± 4.12	43.13 ± 3.38
Sample B	0.32 ± 0.24	26.15 ± 2.46	51.59 ± 5.39	21.94 ± 2.79

^1^ It contained the C–S–H gel phase formed in both the cement matrix and slag rim. ^2^ This value of sample A included the fraction of calcium hydroxide.

**Table 3:** Representative indents from Figure 7 and the corresponding hardness values (GPa).

	Aggregate	Unhydrated Slag Particle	C–S–H		Slag Rim		
Indent	C1R5	C7R6	C6R5	C7R5	C5R6	C9R6	C10R5
Hardness	4.80	3.00	0.62	0.45	0.64	0.96	0.98

## Data Availability

The data presented in this study are available on request from the corresponding author. The data are not publicly available due to privacy.
